# Myofibrillar and Mitochondrial Protein Synthesis Rates Do Not Differ in Young Men Following the Ingestion of Carbohydrate with Whey, Soy, or Leucine-Enriched Soy Protein after Concurrent Resistance- and Endurance-Type Exercise

**DOI:** 10.1093/jn/nxy251

**Published:** 2019-01-29

**Authors:** Tyler A Churchward-Venne, Philippe J M Pinckaers, Joey S J Smeets, Wouter M Peeters, Antoine H Zorenc, Henk Schierbeek, Ian Rollo, Lex B Verdijk, Luc J C van Loon

**Affiliations:** 1 NUTRIM School of Nutrition and Translational Research in Metabolism, Department of Human Biology, Maastricht University Medical Center+, Maastricht, Netherland; 2 Department of Pediatrics, Academic Medical Center, Emma Children's Hospital, Amsterdam, Netherlands; 3 Gatorade Sports Science Institute, Leicester, United Kingdom

**Keywords:** muscle protein synthesis, young men, carbohydrate, dietary protein, whey, soy, leucine, concurrent exercise

## Abstract

**Background:**

Protein ingestion during recovery from resistance-type exercise increases postexercise muscle protein synthesis rates. Whey protein has been reported to have greater anabolic properties than soy protein, an effect which may be attributed to the higher leucine content of whey.

**Objective:**

The objective of this study was to compare postprandial myofibrillar (MyoPS) and mitochondrial (MitoPS) protein synthesis rates after ingestion of carbohydrate with whey, soy, or soy protein enriched with free leucine (to match the leucine content of whey) during recovery from a single bout of concurrent resistance- and endurance-type exercise in young healthy men.

**Methods:**

In a randomized, double-blind, parallel-group design, 36 healthy young recreationally active men (mean ± SEM age: 23 ± 0.4 y) received a primed continuous infusion of l-[ring-^13^C_6_]-phenylalanine and l-[ring-3,5-^2^H_2_]-tyrosine and ingested 45 g carbohydrate with 20 g protein from whey (WHEY), soy (SOY), or leucine-enriched soy (SOY + LEU) after concurrent resistance- and endurance-type exercise. Blood and muscle biopsies were collected over a 360 min postexercise recovery period to assess MyoPS and MitoPS rates, and associated signaling through the mammalian target of rapamycin complex 1 (mTORC1).

**Results:**

Postprandial peak plasma leucine concentrations were significantly higher in WHEY (mean ± SEM: 322 ± 10 μmol/L) and SOY + LEU (328 ± 14 μmol/L) compared with SOY (216 ± 6 μmol/L) (*P* < 0.05). Despite the apparent differences in plasma leucinemia, MyoPS (WHEY: 0.054 ± 0.002; SOY: 0.053 ± 0.004; SOY + LEU: 0.056 ± 0.004%·h^−1^; *P* = 0.83), and MitoPS (WHEY: 0.061 ± 0.004; SOY: 0.061 ± 0.006; SOY + LEU: 0.063 ± 0.004%·h^−1^; *P* = 0.96) rates over the entire 360 min recovery period did not differ between treatments. Similarly, signaling through mTORC1^Ser2448^, p70S6k^Thr389^, 4E-BP1^Thr37/46^, and rpS6^Ser235/236^ was similar between treatments.

**Conclusion:**

Postexercise MyoPS and MitoPS rates do not differ after co-ingestion of carbohydrate with 20 g protein from whey, soy, or leucine-enriched soy protein during 360 min of recovery from concurrent resistance- and endurance-type exercise in young, recreationally active men. This trial was registered at Nederlands Trial Register as NTR5098.

## Introduction

Resistance- (1) and endurance-type (2) exercise increase skeletal muscle protein synthesis (MPS) rates. However, these modes of exercise appear to differentially regulate myofibrillar (MyoPS) and/or mitochondrial (MitoPS) protein subfractional synthetic responses (3, 4). Protein/amino acid intake has been shown to further augment postexercise increases in mixed muscle protein synthesis and MyoPS rates after resistance- (5, 6) and endurance- (7, 8) type exercise, and as such, is commonly recommended to optimize postexercise muscle conditioning (9). However, whether or not protein ingestion can further augment postexercise MitoPS rates has not been established. In addition to ingesting protein to support postexercise muscle conditioning, carbohydrate ingestion is important to facilitate restoration of depleted muscle glycogen stores. Therefore, nutritional strategies to promote exercise recovery generally incorporate the combined intake of carbohydrate and protein (9). Dose-response studies suggest that ingestion of 20 g of a high-quality protein source is sufficient to maximally stimulate MPS rates during recovery from resistance-type exercise (10, 11). In addition, some studies have reported differences in the capacity of various protein sources to stimulate MPS after resistance-type exercise (12–16). Differences in the anabolic properties of various protein sources have been attributed to differences in their digestion and absorption kinetics and/or amino acid content (17). In terms of amino acids, the leucine content of a protein is believed to be of particular importance as leucine has been shown to activate translation initiation (18, 19) and stimulate MPS rates in vivo in humans (20).

Whey and soy protein are nutritionally complete, high-quality proteins (21). Whey protein has relatively high indispensable amino acid (∼50%) and leucine (∼10–12%) contents compared with other proteins (22), and undergoes rapid digestion and absorption after ingestion (23). Soy protein is also regarded as a rapidly digested dietary protein (24), but has lower indispensable amino acid (∼36%) and leucine (∼6%) contents than does whey protein (22). As a consequence, ingestion of soy protein is typically followed by a rapid, but more moderate postprandial rise in plasma amino acid levels (12, 16). Although soy is generally considered a high-quality source of dietary protein, it has been reported to be less effective than whey protein (12, 16) and bovine milk (13) in its capacity to increase MPS rates during recovery from a single bout of resistance-type exercise. This may relate to reported differences in how amino acids derived from soy and milk protein are used. For example, protein-derived amino acid splanchnic retention (24, 25), amino acid catabolism (24, 26), and/or amino acid oxidation (16) all seem greater after ingestion of soy compared to milk-derived proteins. Alternatively, the reduced capacity of soy protein to increase postprandial MPS rates compared to whey protein during recovery from resistance-type exercise may simply be attributed to a lower postprandial rise in leucine concentration (12, 16). In support of this notion, ingestion of a protein blend containing whey, soy, and sodium caseinate has been shown to be as effective as whey protein at stimulating postexercise MPS rates when matched for leucine contents (27). Furthermore, supplementation of soy protein enriched with branched-chain amino acids (leucine, isoleucine, valine) has been reported to enhance whole-body protein synthesis rates (28). Therefore, the capacity of soy protein ingestion to stimulate postexercise MPS rates may be enhanced by addition of free leucine.

The present study examined the effects of co-ingesting 20 g whey protein (WHEY), 20 g soy protein (SOY), or 20 g soy protein enriched with free leucine [to match the total leucine content of whey protein (SOY + LEU)], with 45 g carbohydrate on postprandial MyoPS and MitoPS rates during recovery from a single bout of concurrent resistance- and endurance-type exercise in young, healthy, recreationally active men. We hypothesized that WHEY and SOY + LEU would result in higher postexercise MyoPS and MitoPS rates compared to SOY.

## Methods

### Participants

Thirty-six healthy recreationally active men (age 23 ± 0.4 y; height 1.80 ± 0.01 m; weight 75.0 ± 1.3 kg; values are mean ± SEM) volunteered to participate in this parallel group, double-blind, randomized controlled trial. “Recreationally active” was defined as engaging in sports or structured exercise 1–3 d/wk. Participants’ characteristics are presented in **Table 1**. The characteristics and corresponding data from the whey protein treatment group (WHEY) are also presented in the accompanying manuscript (29). This study was part of a larger trial registered at the Nederlands Trial Register (NTR5098), and was conducted between March 2015 and May 2016 at Maastricht University in Maastricht, Netherlands. All participants were informed of the purpose of the study, the experimental procedures, and possible risks before providing informed written consent to participate. The procedures followed were in accordance with the ethical standards of the medical ethics committee of Maastricht University Medical Centre+ on human experimentation and in accordance with the Helsinki Declaration of 1975 as revised in October 2013. The study was independently monitored by the Clinical Trial Centre Maastricht (CTCM).

**TABLE 1:** Characteristics of young recreationally active men who ingested nutritional treatments consisting of carbohydrate co-ingested with whey, soy, or free leucine-enriched soy protein after a single bout of concurrent exercise^1^

Nutritional treatment group				
	WHEY	SOY	SOY + LEU	*P* value
Age, y	23 ± 1	23 ± 1	23 ± 1	0.98
Height, m	1.80 ± 0.01	1.80 ± 0.02	1.80 ± 0.02	0.97
Weight, kg	76.0 ± 2.0	75.2 ± 2.5	73.8 ± 2.4	0.79
BMI, kg/m^2^	23.4 ± 0.6	23.1 ± 0.6	22.9 ± 0.7	0.81
Fat + bone-free mass, kg	58.6 ± 1.5	59.6 ± 1.8	56.9 ± 1.5	0.50
Fat mass, kg	15.3 ± 0.9	13.6 ± 0.9	14.4 ± 1.0	0.45
% Fat	19.8 ± 0.9	17.7 ± 0.9	19.2 ± 1.0	0.25
Systolic blood pressure, mm Hg	123 ± 3	131 ± 3	123 ± 3	0.10
Diastolic blood pressure, mm Hg	64 ± 4	68 ± 2	66 ± 3	0.62
Leg press 1-RM, kg	271 ± 13	265 ± 16	281 ± 11	0.70
Leg extension 1-RM, kg	121 ± 5	124 ± 7	124 ± 6	0.95
Maximal workload, W	266 ± 13	267 ± 12	267 ± 10	1.00

1 Values are mean ± SEM. *n* = 12 for WHEY, 12 for SOY, and 12 for SOY + LEU. Data were analyzed with use of a 1-factor ANOVA. WHEY, 45 g carbohydrate co-ingested with 20 g whey protein; SOY, 45 g carbohydrate co-ingested with 20 g soy protein; SOY + LEU, 45 g carbohydrate co-ingested with 20 g soy protein enriched with leucine to match the leucine content of WHEY; 1-RM, 1 repetition maximum.

### Preliminary testing

Participants aged 20–30 y inclusive, with a BMI >19.0 and <25.0 (kg/m^2^) underwent an initial screening session to assess height, weight, blood pressure, and body composition (by dual-energy X-ray absorptiometry; Discovery A, Hologic). Participants were deemed healthy based on their responses to a medical questionnaire and screening results. After assessment of baseline anthropometrics, participants were familiarized with the exercise testing protocol and the exercise equipment. All exercise testing during the preliminary testing visit was supervised by >1 of the study investigators. Participants underwent estimates of 1 repetition maximum (1-RM) strength on the supine leg press (Technogym BV) and seated leg extension (Technogym BV) exercise through use of the multiple repetition testing procedure (30). Before testing each exercise, participants performed 10 submaximal repetitions to become familiar with the equipment and to have exercise technique assessed and adjusted by 1 of the study investigators. Working sets were then performed with progressively increased loads until failure to perform a valid estimation within 3–6 repetitions of the set. A repetition was considered valid if the subject was able to complete it in a controlled manner as determined by a study investigator. A 2-min inter-set rest period was allowed between successive sets. After estimates of 1-RM on the leg press and leg extension exercise, peak power output was determined during an incremental test to volitional fatigue on a cycle ergometer (Lode BV). Participants began cycling at a workload equivalent to 2 W/kg bodyweight for 150 s, after which the workload was increased by 25 W every 150 s until volitional fatigue was reached, defined as the inability to maintain a cadence >60 revolutions/min. All equipment settings were noted and replicated during the experimental test day. The pretesting and experimental trials were separated by >5 d.

### Study design

Participants were randomly assigned to ingest a beverage (590 mL) containing 45 g of carbohydrate with 20 g whey protein (WHEY), 20 g soy protein (SOY), or 20 g soy protein supplemented with free leucine to match the leucine content of whey protein (SOY + LEU). The carbohydrate powder was supplied by PepsiCo Inc, and was composed of dextrose and maltodextrin. Whey protein concentrate (Nutri Whey 800F) and soy protein isolate (Unisol DP IP Non GMO) were obtained from FrieslandCampina DMV B.V. and Vitablend Nederland B.V., respectively. The leucine was obtained from Frutarom. Details of the amino acid, protein, and carbohydrate contents of the nutritional treatment are shown in **Table 2**. Random assignment was performed with a computerized list randomizer (https://www.random.org/lists/), and participants were sequentially allocated to a treatment according to the random assignment list.

**TABLE 2:** Amino acid (l-form), protein, and carbohydrate contents of nutritional treatments consisting of carbohydrate co-ingested with whey, soy, or free leucine-enriched soy protein after a single bout of concurrent exercise in young recreationally active men^1^

Nutritional treatment group			
	WHEY	SOY	SOY + LEU
Amino acid content, g			
Alanine	1.02	0.76	0.76
Arginine	0.62	1.34	1.34
Asparagine	2.36	2.04	2.04
Cysteine	0.56	0.22	0.22
Glutamine	3.66	3.36	3.36
Glycine	0.38	0.74	0.74
Histidine	0.44	0.46	0.46
Isoleucine	1.14	0.86	0.86
Leucine	2.58	1.44	1.44
Lysine	2.14	1.12	1.12
Methionine	0.48	0.22	0.22
Phenylalanine	0.78	0.92	0.92
Proline	1.02	0.90	0.90
Serine	0.94	0.92	0.92
Threonine	1.08	0.68	0.68
Tryptophan	0.42	0.24	0.24
Tyrosine	0.74	0.66	0.66
Valine	1.06	0.88	0.88
Added free amino acids, g			
Leucine	0.00	0.00	1.14^2^
Totals, g			
Leucine	2.58	1.44	2.58
ƩNEAA	11.30	10.94	10.94
ƩEAA	10.12	6.82	7.96
ƩAA	21.42	17.76	18.90
Protein^3^	20.00	20.00	20.00
Carbohydrate	45.00	45.00	45.00

1 AA, amino acids; EAA, essential amino acids; NEAA, nonessential amino acids;WHEY, 45 g carbohydrate co-ingested with 20 g whey protein; SOY, 45 g carbohydrate co-ingested with 20 g soy protein; SOY + LEU, 45 g carbohydrate co-ingested with 20 g soy protein enriched with leucine to match the leucine content of WHEY.

2 The added leucine in SOY + LEU was in addition to 20 g total protein.

3 Total protein was calculated as nitrogen × 6.38 for whey and nitrogen × 6.25 for soy protein.

### Diet and physical activity

All participants were instructed to refrain from strenuous physical activity and alcohol consumption for 3 d before the experimental trial. In addition, all participants were instructed to fill out food intake and physical activity questionnaires for 2 d before the experimental trial. On the evening before the experimental trial, all participants consumed a prepackaged standardized meal containing 55% energy as carbohydrate, 30% energy as fat, and 15% energy as protein before 2000, after which they remained fasted.

### Experimental protocol

**FIGURE 1:**
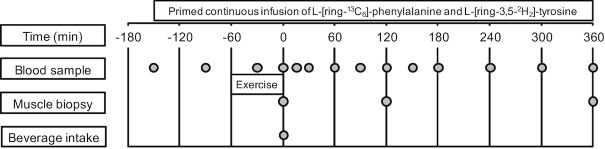
Schematic representation of the experimental design.

At ∼0745, participants arrived at the laboratory in the overnight postabsorptive state. A catheter was inserted into an antecubital vein for stable isotope amino acid infusion, while a second catheter was subsequently inserted into a dorsal hand vein on the contralateral arm for arterialized blood sampling. To obtain arterialized blood samples, the hand was placed in a hot box (60°C) for 10 min before sample collection (31). After taking a baseline blood sample (*t* = −150 min), the plasma phenylalanine pool was primed with a single dose of l-[ring-^13^C_6_]-phenylalanine (2.25 μmol/kg) and l-[ring-3,5-^2^H_2_]-tyrosine (0.867 μmol/kg), and a continuous intravenous infusion of l-[ring-^13^C_6_]-phenylalanine (0.05 μmol kg^−1^ min^−1^) and l-[ring-3,5-^2^H_2_]-tyrosine (0.019 μmol kg^−1^ min^−1^) was initiated (*t* = −150 min) with use of a calibrated IVAC 598 pump. After resting in a supine position for 60 min, a second arterialized blood sample was drawn (*t* = −90 min). After resting for another 30 min, participants initiated (*t* = −60 min) the concurrent exercise intervention (described subsequently). A third blood sample was drawn (*t* = −30 min) during the transition from resistance- to endurance-type exercise. Immediately after the exercise intervention (*t* = 0 min), an arterialized blood sample was drawn and a muscle biopsy sample was collected from the vastus lateralis of a randomly selected leg. Subsequently, participants received a 590 mL beverage corresponding to their randomly assigned treatment allocation [i.e., WHEY (*n* = 12), SOY (*n* = 12), SOY + LEU (*n* = 12)]. The beverages were enriched to 4% l-[ring-^13^C_6_]-phenylalanine to minimize dilution of the steady-state plasma l-[ring-^13^C_6_]-phenylalanine precursor pool implemented by the constant infusion. Arterialized blood samples were then collected at *t* = 15, 30, 60, 90, 120, 150, 180, 240, 300, and 360 min in the postprandial period. Second and third muscle biopsy samples were collected at *t* = 120 and *t* = 360 min to determine postprandial MyoPS and MitoPS rates from *t* = 0–120, 120–360, and 0–360 min. Blood samples were collected into EDTA-containing tubes and centrifuged at 1000 × *g* for 15 min at 4°C. Aliquots of plasma were frozen in liquid nitrogen and stored at −80°C. Biopsy samples were collected with use of a 5-mm Bergström needle custom-adapted for manual suction. Samples were obtained from separate incisions from the middle region of the vastus lateralis, ∼15 cm above the patella and ∼3 cm below entry through the fascia, under 1% xylocaine local anesthesia with adrenaline (1:100,000). Muscle samples were freed from any visible non-muscle material, immediately frozen in liquid nitrogen, and stored at −80°C until further processing. When the experimental protocol was complete, cannulae were removed and subjects were fed and assessed for ∼30 min before leaving the laboratory. For a schematic representation of the infusion protocol, see **Figure 1**.

### Concurrent exercise protocol

#### Resistance-type exercise

Participants began with a standardized warm-up on the supine leg press (1 × 10 repetitions at ∼50% estimated 1-RM), followed by 4 sets of 8 repetitions at ∼80% of their previously estimated 1-RM. Participants then carried out the same exercise protocol (i.e., same number of sets and repetitions at % estimated 1-RM) on the seated leg extension machine. Each set was separated by 2 min of passive recovery during which time the subject remained seated. Range of motion was set from ∼70–155° for the leg press and from ∼75–165° for the leg extension. Strong verbal encouragement was provided by 1 of the study investigators during each set.

#### Endurance-type exercise

After the resistance-type exercise, participants performed 30 min of continuous cycling at ∼60% of their previously determined maximal workload (W_max_). Participants were allowed ad libitum access to water during cycling. Visual feedback for pedal frequency and elapsed time were provided to participants and strong verbal encouragement was provided by 1 of the study investigators.

### Plasma and muscle tissue analyses

#### Plasma analyses

Details of analysis relating to the determination of plasma glucose, insulin, and amino acid concentrations as well as plasma l-[ring-^13^C_6_]-phenylalanine, l-[ring-^13^C_6_]-tyrosine, and l-[ring-3,5-^2^H_2_]-tyrosine enrichments are presented in **Supplemental Methods**.

#### Muscle tissue analyses

A piece of wet muscle (∼100 mg) was homogenized on ice with use of a Teflon pestle in ice-cold homogenization buffer (10 μL/mg; 1 M sucrose, 1 M Tris/HCl, 1 M KCl, 1 M EDTA) containing protease/phosphatase inhibitor cocktail tablets (Complete Protease Inhibitor Mini-Tabs; and PhosSTOP, Roche Applied Science). After ∼5–10 min of hand homogenization, the homogenate was centrifuged at 700 ×*g* for 15 min at 4°C to pellet a myofibrillar protein-enriched fraction. The supernatant was transferred to another tube and centrifuged at 12,000 × *g* for 20 min at 4°C to pellet a mitochondrial protein-enriched fraction. The resulting supernatant was used for Western Blot analysis. Additional details regarding the preparation and analysis of skeletal muscle samples for measurement of myofibrillar and mitochondrial protein-bound phenylalanine enrichment, and intramuscular signaling via Western Blot are presented in **Supplementary Methods**.

## Calculations

The FSR of myofibrillar and mitochondrial protein enriched fractions was calculated through use of the standard precursor-product equation

(1) \begin{equation*} {\rm{FSR}} = [({E_{2{\rm{b}}}} - {E_{1{\rm{b}}}})/({E_{{\rm{precursor}}}} \times t)] \times 100 \end{equation*}

where *E*b is the increment in myofibrillar or mitochondrial protein-bound l-[ring-^13^C_6_]-phenylalanine enrichment mole % excess (MPE) between 2 muscle biopsy samples, E_precursor_ is the weighted mean plasma l-[ring-^13^C_6_]-phenylalanine enrichment (MPE) during the tracer incorporation period, and *t* is the tracer incorporation time in h. Weighted mean plasma enrichments were calculated by taking the measured enrichment between consecutive time points and correcting for the time between these sampling time points. For calculation of postprandial FSR, biopsy samples at *t* = 0, 120, and 360 min were used.

### Statistical analysis

Subjects’ characteristics, 1-RM strength, and W_max_ data were analyzed with use of a 1-factor (treatment) ANOVA. Blood glucose and plasma insulin were analyzed with use of a 2-factor (treatment × time) repeated-measures ANOVA. Plasma leucine, phenylalanine, and tyrosine concentrations were analyzed with use of a 2-factor (treatment × time) repeated-measures ANOVA. Leucine, phenylalanine, and tyrosine AUC was analyzed with use of a 1-factor (treatment) ANOVA. Plasma enrichments were analyzed with use of a 2-factor (treatment × time) repeated-measures ANOVA. Myofibrillar and mitochondrial FSR during early and late recovery (i.e., 0–120 and 120–360 min) and protein phosphorylation status (i.e., 0, 120, and 360 min) were analyzed with use of a 2-factor (treatment × time) repeated-measures ANOVA. The aggregate myofibrillar and mitochondrial FSR (i.e., 0–360 min) was analyzed with use of a 1-factor (treatment) ANOVA. A power calculation was performed with differences in postprandial myofibrillar protein FSR as the primary outcome measure with the use of a standard deviation of 0.0065%·h^−1^ in all treatments, and a difference in FSR of 0.008%·h^−1^ between treatments (or ∼20% when expressed as relative difference between treatments). With a power of 80% and a significance level of 0.05, the final number of participants to be included was calculated as *n* = 12 per group. Tukey's post hoc analysis was performed whenever a significant F ratio was found to isolate specific differences. Statistical analyses were performed with a software package (IBM SPSS Statistics for Windows, version 21.0, IBM Corp.). Means were considered to be significantly different for *P* values <0.05.

## Results

### Plasma analyses

**FIGURE 2:**
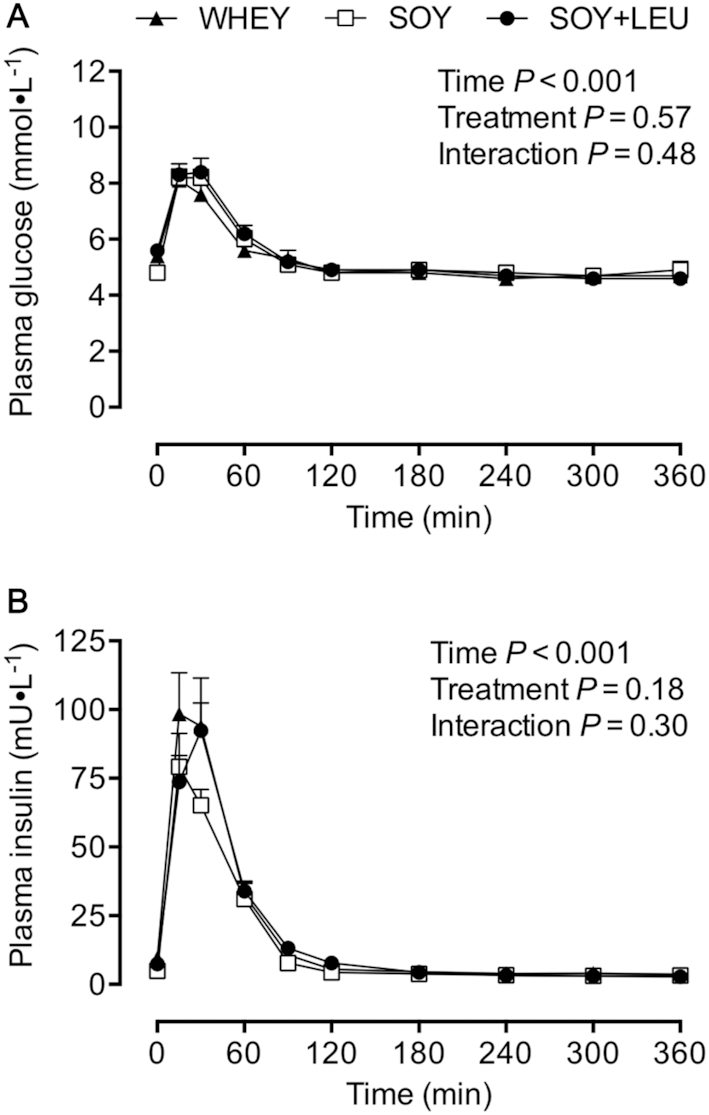
Plasma glucose (A) and insulin (B) concentrations during postabsorptive conditions (*t* = 0 min), and during postprandial conditions (*t* = 15–360 min) after beverage intake during recovery from a single bout of concurrent exercise in young men. Data for glucose and insulin were analyzed with use of a 2-factor repeated measures ANOVA. Values are mean ± SEM. *n* = 12. WHEY, 45 g carbohydrate co-ingested with 20 g whey protein; SOY, 45 g carbohydrate co-ingested with 20 g soy protein; SOY + LEU, 45 g carbohydrate co-ingested with 20 g soy protein enriched with leucine to match the leucine content of WHEY.

**FIGURE 3:**
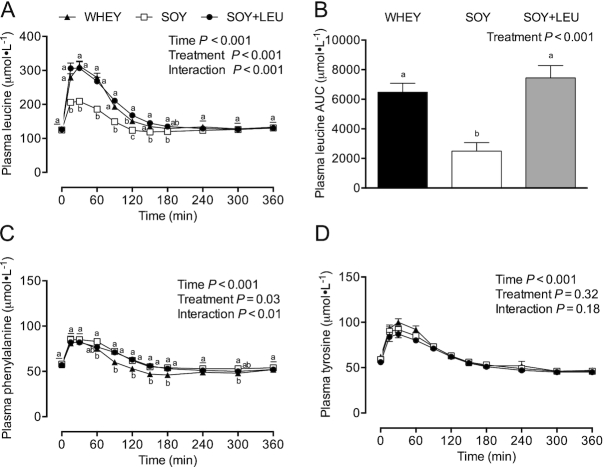
Plasma leucine (A), leucine AUC (B), phenylalanine (C), and tyrosine (D) concentrations during postabsorptive conditions (*t* = 0 min), and during postprandial conditions (*t* = 15–360 min) after beverage intake during recovery from a single bout of concurrent exercise in young men. Data for leucine, phenylalanine, and tyrosine were analyzed with use of a 2-factor repeated measures ANOVA. Data for leucine AUC were analyzed with use of a 1-factor ANOVA. Values are mean ± SEM. *n* = 12. Labeled means within a time without a common letter differ, *P* < 0.05. WHEY, 45 g carbohydrate co-ingested with 20 g whey protein; SOY, 45 g carbohydrate co-ingested with 20 g soy protein; SOY + LEU, 45 g carbohydrate co-ingested with 20 g soy protein enriched with leucine to match the leucine content of WHEY.

Plasma glucose concentrations (**Figure 2**A) were transiently increased from *t* = 15–60 min after ingestion of the protein-carbohydrate containing treatment beverages (*P* < 0.001). Similarly, plasma insulin concentrations (Figure 2B) were increased during the postprandial period (*P* < 0.001) from *t* = 15–90 min after beverage intake. Plasma leucine concentrations (**Figure 3**A) were increased in each treatment group during the postprandial period, with WHEY (from *t* = 15–150 min) and SOY + LEU (from *t* = 15–180 min) resulting in higher leucine concentrations when compared with SOY (*P*-interaction < 0.001). Plasma leucine AUC (Figure 3B) over the 360 min postprandial period was greater in WHEY and SOY + LEU compared with SOY (*P* < 0.001). Plasma phenylalanine concentrations (Figure 3C) were transiently increased after protein-carbohydrate co-ingestion, with higher concentrations in response to SOY at *t* = 60 and *t* = 300 min, and both SOY and SOY + LEU from *t* = 90–180 min compared with WHEY during the postprandial period (*P*-interaction < 0.01). Plasma phenylalanine AUC (data not shown) was greater in SOY and SOY + LEU compared with WHEY (*P* = 0.003). Plasma tyrosine concentrations (Figure 3D) were increased from *t* = 15–120 min during the postprandial period (*P* < 0.001). Plasma tyrosine AUC (data not shown) did not differ between treatments (*P* = 0.68).

### Stable isotope tracer analyses

**FIGURE 4:**
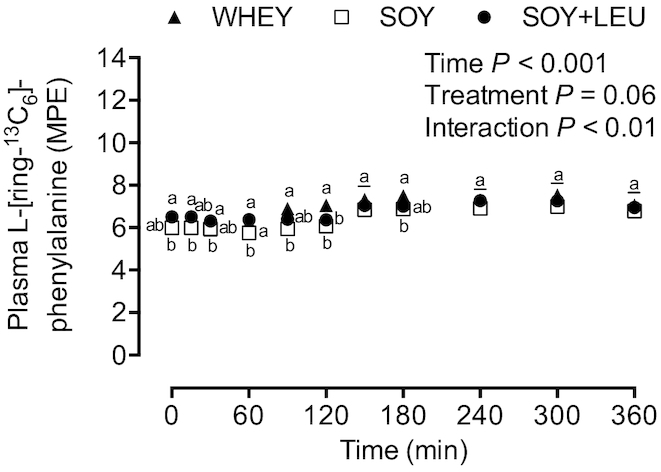
Plasma l-[ring-^13^C_6_]-phenylalanine enrichments during postabsorptive conditions (*t* = 0 min), and during postprandial conditions (*t* = 15–360 min) after beverage intake during recovery from a single bout of concurrent exercise in young men. Data were analyzed with use of a 2-factor repeated measures ANOVA. Values are mean ± SEM. *n* = 12. Labeled means within a time without a common letter differ, *P* < 0.05. MPE, mole % excess; WHEY, 45 g carbohydrate co-ingested with 20 g whey protein; SOY, 45 g carbohydrate co-ingested with 20 g soy protein; SOY + LEU, 45 g carbohydrate co-ingested with 20 g soy protein enriched with leucine to match the leucine content of WHEY.

Plasma l-[ring-^13^C_6_]-phenylalanine enrichments (**Figure 4**) were different between SOY and SOY + LEU at *t* = 0, 15, and 60 min, different between WHEY and SOY + LEU at *t* = 30 and 120 min, and different between WHEY and SOY at *t* = 60, 90, 120, and 180 min (*P*-interaction < 0.01).

**FIGURE 5:**
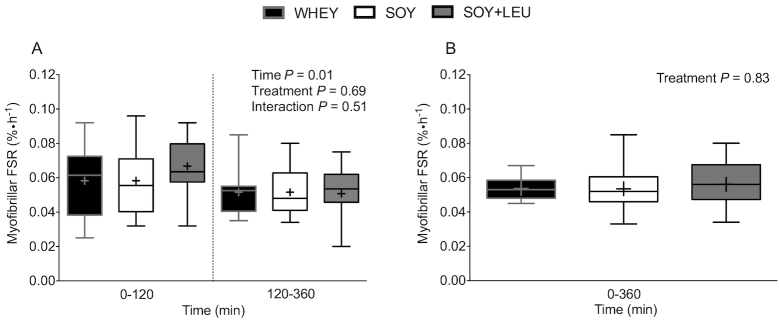
Myofibrillar protein FSR over 0–120 and 120–360 min (A), and over 0–360 min (B) after beverage intake during recovery from a single bout of concurrent exercise in young men. Time-course (A) data were analyzed with use of a 2-factor repeated measures ANOVA. Aggregate (B) data were analyzed with use of a 1-factor ANOVA. Boxes represent 25th to 75th percentiles. Horizontal lines and crosses within boxes represent medians and means, respectively. Whiskers represent minimums and maximums. *n* = 12. FSR, fractional synthetic rate; WHEY, 45 g carbohydrate co-ingested with 20 g whey protein; SOY, 45 g carbohydrate co-ingested with 20 g soy protein; SOY + LEU, 45 g carbohydrate co-ingested with 20 g soy protein enriched with leucine to match the leucine content of WHEY.

**FIGURE 6:**
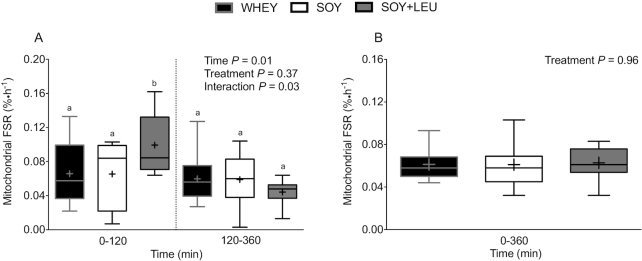
Mitochondrial protein FSR over 0–120 and 120–360 min (A), and over 0–360 min (B) after beverage intake during recovery from a single bout of concurrent exercise in young men. Time-course (A) data were analyzed with use of a 2-factor repeated measures ANOVA. Aggregate (B) data were analyzed with use of a 1-factor ANOVA. Boxes represent 25th to 75th percentiles. Horizontal lines and crosses within boxes represent medians and means, respectively. Whiskers represent minimums and maximums. *n* = 12 for WHEY, 11 for SOY, and 12 for SOY + LEU. Labeled means within a time without a common letter differ, *P* < 0.05. FSR, fractional synthetic rate; WHEY, 45 g carbohydrate co-ingested with 20 g whey protein; SOY, 45 g carbohydrate co-ingested with 20 g soy protein; SOY + LEU, 45 g carbohydrate co-ingested with 20 g soy protein enriched with leucine to match the leucine content of WHEY.

Postprandial MyoPS rates (i.e., myofibrillar FSR), assessed during early (0–120 min) and late (120–360 min) postexercise recovery (**Figure 5**A), did not differ between treatments (*P* = 0.69). MyoPS rates were higher during early (0–120 min) compared with late (120–360 min) postexercise recovery (*P* = 0.01). Aggregate (i.e., 0–360 min) MyoPS rates (Figure 5B) did not differ between treatments (*P* = 0.83). Postprandial MitoPS rates (i.e., mitochondrial FSR), assessed during early (0–120 min) and late (120–360 min) postexercise recovery (**Figure 6**A), were greater in SOY + LEU compared to WHEY and SOY during early, but not late, postexercise recovery (*P*-interaction = 0.03). Aggregate (i.e., 0–360 min) MitoPS rates (Figure 6B) did not differ between treatments (*P* = 0.96).

### Muscle tissue signaling

**FIGURE 7:**
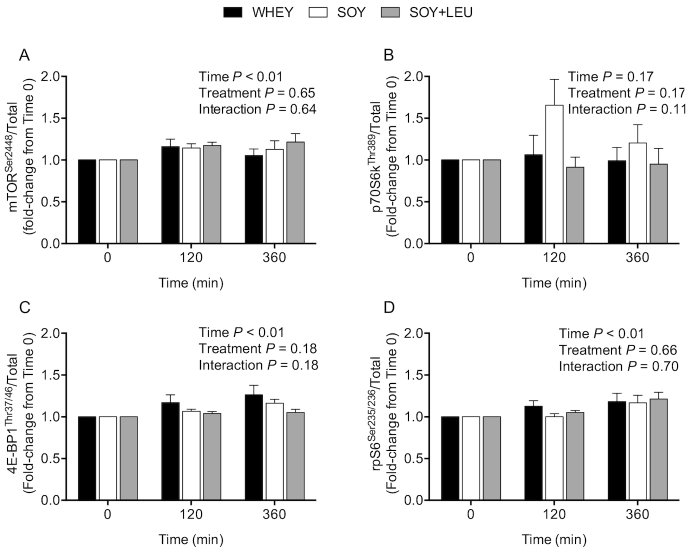
Phosphorylation of mTOR^Ser2448^ (A), p70S6k^Thr389^ (B), 4E-BP1^Thr37/46^ (C), and rpS6^Ser235/236^ (D) relative to the total abundance of their corresponding protein during postabsorptive conditions (*t* = 0 min), and during postprandial conditions (*t* = 120 and 360 min) after beverage intake during recovery from a single bout of concurrent exercise in young men. Data at *t* = 120 min and *t* = 360 min are expressed as fold-change from *t* = 0 min. Data were analyzed with use of a 2-factor repeated measures ANOVA. Values are mean ± SEM. *n* = 12. mTOR, mammalian target of rapamycin; p70S6k, ribosomal protein S6 kinase; rpS6, ribosomal protein S6; WHEY, 45 g carbohydrate co-ingested with 20 g whey protein; SOY, 45 g carbohydrate co-ingested with 20 g soy protein; SOY + LEU, 45 g carbohydrate co-ingested with 20 g soy protein enriched with leucine to match the leucine content of WHEY; 4E-BP1, eukaryotic initiation factor 4E binding protein 1.

**FIGURE 8:**
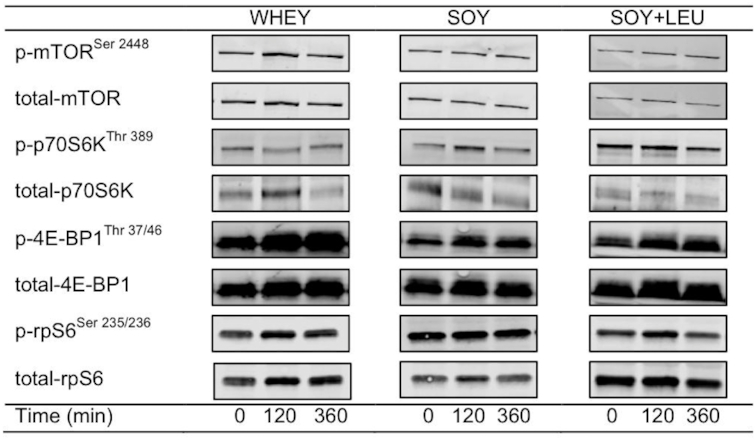
Representative Western Blot images for phosphorylated (p) and total mammalian target of rapamycin (mTOR^Ser2448^), ribosomal protein S6 kinase (p70S6k^Thr389^), eukaryotic initiation factor 4E binding protein 1 (4E-BP1^Thr37/46^), and ribosomal protein S6 (rps6^Ser235/236^) during postabsorptive conditions (*t* = 0 min), and during postprandial conditions (*t* = 120 and 360 min) after beverage intake during recovery from a single bout of concurrent exercise in young men. WHEY, 45 g carbohydrate co-ingested with 20 g whey protein; SOY, 45 g carbohydrate co-ingested with 20 g soy protein; SOY + LEU, 45 g carbohydrate co-ingested with 20 g soy protein enriched with leucine to match the leucine content of WHEY.

The phosphorylation status of mTOR^Ser2448^ (**Figure 7A**) was not different between treatments (*P* = 0.65), but was increased during the postprandial period after concurrent exercise at both *t* = 120 min and *t* = 360 min (*P* < 0.01). The phosphorylation status of p70S6k (ribosomal protein S6 kinase)^Thr389^ (Figure 7B) was not different between treatments (*P* = 0.17), and was not increased during the postprandial period after concurrent exercise (*P* = 0.17). 4E-BP1 (eukaryotic initiation factor 4E binding protein 1)^Thr37/46^ phosphorylation (Figure 7C) was increased during the postprandial period after concurrent exercise at *t* = 120 min, and more so at *t* = 360 min when compared to *t* = 0 min (*P* < 0.01), with no differences between treatments (*P* = 0.18). The phosphorylation of rpS6 (ribosomal protein S6)^Ser235/236^ (Figure 7D) was increased at *t* = 120 min, and more so at *t* = 360 min during the postprandial period after concurrent exercise when compared to *t* = 0 min (*P* < 0.01), with no differences between treatments (*P* = 0.66). Representative Western Blot images are shown in **Figure 8**.

## Discussion

In the present study, we did not observe higher MyoPS rates during recovery from concurrent resistance- and endurance-type exercise after co-ingestion of 45 g of carbohydrate with 20 g whey (WHEY) or 20 g soy protein enriched with free leucine (SOY + LEU) when compared with 20 g soy protein (SOY). This lack of difference was observed despite substantially greater postprandial plasma leucine concentrations after WHEY and SOY + LEU when compared with SOY. Similarly, although we observed greater MitoPS rates during early (*t* = 0–120 min) recovery from concurrent exercise after SOY + LEU when compared to WHEY and SOY, late (*t* = 120–360 min) and overall aggregate (*t* = 0–360 min) MitoPS rates did not differ among treatments.

Ingestion of dietary protein and exercise (i.e., skeletal muscle contraction) represent 2 of the most potent regulators of skeletal muscle protein metabolism (32). The increase in postprandial amino acid availability after protein ingestion results in a marked stimulation of MPS rates (for review see (33)). The postprandial increase in MPS rates is attributed to an increase in circulating indispensable amino acids (IAAs) (34), with leucine (20) being of particular relevance. The postprandial rise in dispensable amino acid concentrations appears to be of less relevance to the stimulation of MPS rates (35). The increase in MPS rates in response to amino acids is further augmented by prior resistance-type exercise (6). Studies examining the dose-dependent relationship between protein ingestion and MPS rates after resistance-type exercise in young adults have demonstrated that ingestion of ∼20 g of a high-quality protein source is sufficient to maximize postexercise mixed muscle protein synthesis (11) and MyoPS (10) rates. Ingestion of more protein does not further stimulate protein synthesis, but is instead oxidized (11) and directed towards ureagenesis (10). As such, in the present study we provided subjects with 20 g whey or soy protein to compare these protein sources in their capacity to support MyoPS and MitoPS rates during recovery from a single bout of combined resistance- and endurance-type exercise (i.e., concurrent exercise).

Whey protein is among the highest quality sources of dietary protein because of its amino acid profile (high IAA, branched-chain amino acid, and leucine contents), rapid digestibility, and robust capacity to stimulate postprandial MPS rates (22). Soy protein is also a rapidly digested high-quality protein source with a favorable IAA content, but ingestion of soy protein has been reported to be less effective in stimulating MPS rates when compared with the ingestion of whey (12, 16) and bovine milk (13) protein during recovery from resistance-type exercise. The lower capacity of soy protein to stimulate postexercise MPS rates when compared to whey may be attributed to its low(er) leucine content (6–8% compared with 10–12% leucine). As such, we also examined the impact of ingesting soy protein enriched with free leucine (to match that of whey protein) on postexercise MyoPS and MitoPS rates. Peak plasma leucine concentrations were considerably higher after WHEY (322 ± 10 μmol/L; ∼152 ± 10% increase) when compared to SOY (216 ± 6 μmol/L; ∼75 ± 7% increase) protein. Fortification of soy protein with free leucine (SOY + LEU) increased postprandial peak leucine concentrations substantially, with levels similar to WHEY (328 ± 14 μmol/L; ∼165 ± 15% increase). In agreement, total plasma leucine exposure (AUC) during the entire 6 h (360 min) recovery period was greater after WHEY and SOY + LEU when compared with SOY (Figure 3B). Despite the differences in postprandial plasma leucinemia between treatments (Figure 3A and B), we observed no differences in MyoPS rates after WHEY, SOY, or SOY + LEU when assessed during the early (from 0–120 min), late (120–360 min), or aggregate 6 h (0–360 min) postexercise recovery periods (Figure 5A and B). Similarly, aside from greater early MitoPS rates after SOY + LEU, we observed no differences in MitoPS rates among treatments.

Based on previous research comparing postexercise MPS rates after ingestion of soy compared to whey (12, 16) or milk (13) protein, we hypothesized that WHEY and SOY + LEU would result in higher MyoPS and MitoPS rates when compared with SOY. Previous research comparing the postprandial metabolic use of dietary nitrogen from soy and total milk protein demonstrated that milk protein better supported “peripheral” protein synthesis (i.e., skeletal muscle) whereas soy protein better stimulated splanchnic protein synthesis rates when assessed under resting conditions (24, 25). Subsequent work from Wilkinson and colleagues (13) demonstrated that ingestion of milk (∼18 g protein) resulted in a more positive amino acid balance across the leg and greater MPS rates when compared to the ingestion of an isonitrogenous, isoenergetic, and macronutrient composition-matched amount of soy protein during recovery from resistance-type exercise. It was proposed that the attenuated postprandial rise in protein-derived amino acid availability after milk as opposed to soy protein ingestion may have been responsible for the greater leg amino acid uptake and higher MPS rates during recovery from resistance-type exercise (13). However, later work from Tang and colleagues (12) demonstrated that more rapidly digested whey protein resulted in higher postprandial MPS rates when compared with the ingestion of soy and micellar casein protein. Whereas micellar casein has been classified as a more slowly digested dietary protein (23), the protein digestion and amino acid absorption kinetics of soy protein seem to be more similar to whey (25). Therefore, potential differences in the anabolic properties of soy compared with whey protein ingestion during postexercise recovery are unlikely to be related to differences in protein digestion and amino acid absorption kinetics and may be more related to differences in amino acid composition of the proteins. The IAA and leucine contents of whey protein are considerably greater than those of soy protein (36). Leucine is a major nutrient regulator of translation initiation (18, 19) that can stimulate MPS rates in humans (20). Supplementing soy protein with BCAAs (leucine, isoleucine, and valine) has also been reported to increase its postprandial anabolic properties in elderly and clinical populations (28). Consequently, we hypothesized that fortifying 20 g soy protein with free leucine (SOY + LEU) to match the leucine content of 20 g whey protein would increase its anabolic properties and augment postprandial MyoPS and MitoPS rates when compared to the ingestion of soy only (SOY). In contrast to our hypotheses, we did not detect any differences in postprandial MyoPS rates after WHEY, SOY, or SOY + LEU during the early (0–120 min), late (120–360 min), as well as aggregate (0–360 min) periods of recovery after concurrent exercise. Similarly, aside from greater postprandial MitoPS rates after SOY + LEU during the early (0–120 min) period of recovery after concurrent exercise, MitoPS rates did not differ between treatments over the late (120–360 min) or aggregate (0–360 min) postexercise recovery periods.

The absence of differences in MyoPS rates during recovery from concurrent exercise after ingestion of 20 g whey, soy, or free leucine-enriched soy, implies that exercise and/or nutrient signals that regulate postexercise MyoPS rates were equivalent between treatment groups. Because the current study did not incorporate a nonprotein control treatment, we cannot determine the independent contribution of whey or soy protein ingestion to MyoPS rates during recovery from concurrent exercise. In an accompanying study (29), we show that co-ingestion of 20 g protein with 45 g carbohydrate resulted in only marginal increases in MyoPS rates (∼16%) when compared to the ingestion of 45 g carbohydrate only during recovery from concurrent exercise. Ingested carbohydrate and the associated increase in insulin availability may have stimulated increases in blood flow (37), microvascular perfusion (38), amino acid transport (39), and/or a suppression of protein degradation in skeletal muscle tissue (39). In addition, carbohydrate co-ingestion with protein may have delayed the digestion and absorption kinetics of the ingested proteins (40). As such, potential differences in MyoPS rates after ingestion of various sources of isolated dietary protein may be less apparent when co-ingested with carbohydrate. Consequently, greater doses of protein (e.g., 30 g) may be required to robustly increase MyoPS rates when protein is co-ingested with carbohydrate during recovery from concurrent exercise and, as such, may be necessary to reveal any differences in the anabolic properties of ingested whey, soy, or free leucine-enriched soy after exercise.

Currently, there is limited information available on the nutritional regulation of MitoPS in human muscle. Early studies demonstrated that amino acid provision via intravenous infusion may stimulate MitoPS rates at rest (41, 42). More recent studies have demonstrated that orally ingested protein can increase MitoPS rates at rest (43, 44), and that the source of ingested protein may modulate the response of MitoPS (44). However, studies to date evaluating the effect of protein ingestion on MitoPS rates in humans have failed to find support for the notion that protein ingestion positively augments MitoPS rates after exercise (7, 45–47). The results of the present study, and those reported in our accompanying study (29), are the first to report the effect of different sources of ingested protein on postprandial MitoPS after exercise. The reason for the greater increase in early (0–120 min) postprandial MitoPS rates after SOY + LEU in the current study is unclear, but may be a result of differences in the metabolism of free versus protein-bound leucine. Leucine has been reported to increase mitochondrial content and mitochondrial biogenesis-related gene expression in C2C12 myotubes (48, 49). Nonetheless, the effect was short-lived as MitoPS rates did not differ between treatments over the late (120–360 min) or aggregate (0–360 min) postexercise recovery periods.

In line with the absence of any major differences in MyoPS or MitoPS rates after WHEY, SOY, or SOY + LEU during recovery from concurrent exercise, we did not detect any differences in the phosphorylation status of the intracellular signaling proteins between treatments (Figure 7A–D). To date, few studies have compared signaling responses of protein targets of the mTORC1 pathway after ingestion of different sources of dietary protein during postexercise recovery (50–53). Anthony and colleagues (50) reported that rats fed carbohydrate plus whey protein demonstrated greater increases in mTOR^Ser2448^ and p70S6k^Thr389^ phosphorylation compared to rats fed carbohydrate and soy protein after treadmill exercise (50). In accordance, Mitchell and colleagues (51) reported that ingestion of 30 g soy protein was less effective than whey protein at sustaining postprandial increases in p70S6k^Thr389^ phosphorylation during recovery from resistance-type exercise in older adults. The reason for the lack of differences in signaling responses in mTOR^Ser2448^, p70S6k^Thr389^, 4E-BP1^Thr37/46^, and rpS6^Ser235/236^ after ingestion of whey, soy, or free leucine-enriched soy protein despite substantial differences in plasma leucine availability remains unclear but may relate to the timing of biopsy sampling (i.e., at 120 and 360 min during the postprandial period) as well as the postexercise conditions in which these measurements were performed. A bout of concurrent exercise may have already increased amino acid availability to the muscle by increasing endogenous amino acid release, and/or stimulating skeletal muscle blood flow. These factors may have made the contribution of the postprandial release of exogenous amino acids less relevant to the changes in mTORC1 and its downstream targets. Alternatively, as peak signaling responses via mTORC1 and/or its downstream targets may occur early after protein intake (54), we may have missed divergent signaling responses among protein treatments that occurred before the biopsy taken at 120 minutes into the postprandial period after concurrent exercise. Either way, the lack of differences in myocellular signaling responses among treatments are in line with the absence of major differences in postexercise MyoPS and MitoPS rates.

In conclusion, co-ingestion of carbohydrate with 20 g whey, soy, or free leucine-enriched soy protein do not lead to differences in overall postexercise MyoPS or MitoPS rates during recovery from a single bout of concurrent resistance- and endurance-type exercise in recreationally active young men. Increasing the leucine content of soy protein to match that of whey protein does not further increase postexercise MyoPS or MitoPS rates during 360 min of recovery from concurrent resistance- and endurance-type exercise.

## Supplementary Material

nxy251_Supplemental_File

## Notes

Supported by PepsiCo/Gatorade Sports Science Institute.

Supplemental methods are available from the “Supplementary data” link in the online posting of the article and from the same link in the online table of contents at https://academic.oup.com/jn/.

Abbreviations used: 4E-BP1, eukaryotic initiation factor 4E binding protein 1; FSR, fractional synthetic rate; IAA, indispensable amino acids; MitoPS, mitochondrial protein synthesis; MPE, mole % excess; MPS, muscle protein synthesis; mTORC1, mammalian target of rapamycin complex 1; MyoPS, myofibrillar protein synthesis; p70S6k, ribosomal protein S6 kinase; rpS6, ribosomal protein S6; SOY, 45 g carbohydrate co-ingested with 20 g soy protein; SOY + LEU, 45 g carbohydrate co-ingested with 20 g soy protein enriched with leucine to match the leucine content of WHEY; WHEY, 45 g carbohydrate co-ingested with 20 g whey protein; 1-RM, 1 repetition maximum.
